# Utility of Machine Learning Models to Predict Lymph Node Metastasis of Japanese Localized Prostate Cancer

**DOI:** 10.3390/cancers16234073

**Published:** 2024-12-05

**Authors:** Hideto Ueki, Tomoaki Terakawa, Takuto Hara, Munenori Uemura, Yasuyoshi Okamura, Kotaro Suzuki, Yukari Bando, Jun Teishima, Yuzo Nakano, Raizo Yamaguchi, Hideaki Miyake

**Affiliations:** 1 Department of Urology, Kobe University Graduate School of Medicine, Kobe 650-0017, Japan; hideueki@med.kobe-u.ac.jp (H.U.); supermarimo85@gmail.com (T.H.); dfgky008@gmail.com (Y.O.); pikataro1012@gmail.com (K.S.); yukaribando@hotmail.co.jp (Y.B.); teishima@med.kobe-u.ac.jp (J.T.); yznakano@med.kobe-u.ac.jp (Y.N.); hmiyake@med.kobe-u.ac.jp (H.M.); 2 Department of International Clinical Cancer Research and Promotion, Kobe University Graduate School of Medicine, Kobe 650-0047, Japan; munenori@med.kobe-u.ac.jp; 3 Department of Urology, Kobe University Hospital International Clinical Cancer Research Center, Kobe 650-0047, Japan; raizoy@med.kobe-u.ac.jp

**Keywords:** lymph node metastasis, machine learning, prostate cancer

## Abstract

Background/Objectives: Extended pelvic lymph node dissection is a crucial surgical technique for managing intermediate to high-risk prostate cancer. Accurately predicting lymph node metastasis before surgery can minimize unnecessary lymph node dissections and their associated complications. This study assessed the efficacy of various machine learning models for predicting lymph node metastasis in a cohort of Japanese patients who underwent robot-assisted laparoscopic radical prostatectomy. Methods: Data from 625 patients who underwent extended pelvic lymph node dissection or standard dissection with lymph node metastasis between October 2010 and February 2023 were analyzed. Four machine learning models—Random Forest, Light Gradient-Boosting Machine, Logistic Regression, and Support Vector Machine—were used to predict lymph node metastasis. Their performance was assessed using receiver operating characteristic curves, a decision curve analysis, and predictive values at different thresholds. Results: Lymph node metastasis was observed in 34 patients (5.4%). The Light Gradient-Boosting Machine had the highest AUC of 0.924, followed by the Random Forest model with an AUC of 0.894. The decision curve analysis indicated substantial net benefits for both models, particularly at low threshold probabilities. The Light Gradient-Boosting Machine demonstrated superior accuracy, achieving 95.6% at the 0.05 threshold and 96.7% at the 0.10 threshold, outperforming other models and conventional nomograms in the validation dataset. Conclusion: Machine learning models, especially Light Gradient-Boosting Machine and Random Forest, show significant potential for predicting lymph node metastasis in prostate cancer, thereby aiding in reducing unnecessary surgical interventions.

## Simple Summary

In this study, machine learning models were developed to predict lymph node invasion in Japanese prostate cancer patients, using a structured approach to improve predictive accuracy. Traditional methods, such as the Briganti nomogram, have been widely used but may lack flexibility and adaptability to diverse clinical datasets. Our machine learning models, especially Light Gradient-Boosting Machine and Random Forest, showed higher flexibility and accuracy compared to conventional models. Through a thorough data analysis and feature engineering, including the use of predictive features such as PSA levels and tumor stage, we enhanced model performance and interpretability. A decision curve analysis indicated significant clinical benefits, suggesting that these models can better identify candidates for lymph node dissection, thereby minimizing unnecessary procedures. However, validation with larger and multi-institutional datasets is needed to confirm the utility of these models in broader clinical settings.

## 1. Introduction

Extended pelvic lymph node dissection (ePLND) remains an integral part of radical prostatectomy (RP) [1,2,3], and is an option for patients with intermediate and high-risk prostate cancer [4,5,6]. To identify appropriate cases for ePLND, The European Association of Urology and National Comprehensive Cancer Network (NCCN) guidelines recommend the use of predictive models such as the Briganti [5] and the Memorial Sloan Kettering Cancer Center (MSKCC) [7] nomograms. The Briganti nomogram incorporates the preoperative prostate-specific antigen (PSA), biopsy Gleason grade group, and percentage of positive cores to estimate the risk of lymph node involvement (LNI). It is widely used for predicting LNI and helps select those patients who should be considered for ePLND in clinical practice [8]. The MSKCC nomogram builds upon these variables by including additional patient-specific factors, such as age, to predict a range of prostate cancer outcomes, including biochemical recurrence and LNI. However, both models rely on regression-based methods, which assume linear relationships between variables and fail to effectively capture complex, non-linear interactions. These limitations result in suboptimal predictive accuracy and can lead to overtreatment, with some patients undergoing unnecessary ePLND. Overtreatment is a significant concern in prostate cancer management. A recent meta-analysis demonstrated that perioperative complications associated with ePLND are significantly more frequent compared to limited pelvic lymph node dissection (PLND) [9]. Therefore, ongoing research aims to improve prediction methods to minimize unnecessary ePLND and its associated complications.

From a clinical perspective, achieving an optimal balance between sensitivity and specificity in identifying candidates for ePLND is crucial [3]. Several nomograms are available to urologic surgeons to predict pelvic LNI of prostate cancer from pre-surgical data, including MRI findings, but difficulties remain about how best to integrate imaging modalities with clinical and biopsy variables to enable LNI to be predicted accurately.

Recently, scientists have made great efforts to explore different methods for more accurately evaluating the risks of LNI, and machine learning (ML) has become a powerful tool for improving clinical strategies in medical research [10,11,12]. Compared with a traditional regression analysis, ML algorithms have significant advantages in prediction performance in large databases [10,13]. Unlike regression-based nomograms, ML algorithms can learn non-linear patterns and interactions between clinical variables, making them particularly suitable for prostate cancer risk prediction. Despite these advantages, there is currently no effective ML model specifically tailored for predicting the risk of LNI in Japanese patients undergoing RP. 

In this study, we developed ML models to predict the risk of pelvic LNI from prostate cancer in Japanese patients who underwent RP. Additionally, these models were compared to conventional predictive tools, such as the Briganti and MSKCC nomograms, to evaluate whether ML approaches could address their limitations and improve predictive accuracy. The main contributions of this study are as follows:

● We developed ML models specifically tailored for Japanese patients to address the lack of existing ML models tailored for this population, enabling more accurate and personalized predictions for LNI.
● The predictive performance of ML models was systematically evaluated in comparison to widely used nomograms, highlighting their potential to improve LNI prediction.
● The capability of ML models to handle complex, non-linear relationships among clinical variables was demonstrated, offering a significant advantage over traditional regression-based approaches.
● Key predictive features for LNI were identified, which may serve as a foundation for further advancements in prostate cancer research and clinical practice.

## 2. Materials and Methods

### 2.1. Study Population

A total of 803 patients who underwent robot-assisted laparoscopic radical prostatectomy (RARP) between October 2010 and February 2023 were retrospectively identified. Of these, 759 surgeries were performed at Kobe University Hospital, and 44 surgeries were performed at the International Clinical Cancer Research Center. Among these, 60 patients who received neoadjuvant hormonal therapy and 118 patients who underwent RARP without PLND were excluded. A total of 625 patients were included in the final analysis for the development and validation of the ML models. Biopsies were performed under US guidance and typically 12–16 cores were taken. More cores were taken in cases in which re-biopsies or targeted biopsies were performed. All specimens were submitted for pathologic evaluation in multiple packages and were evaluated by pathologists [5]. Extended PLND was performed for high-risk patients with prostate cancer according to the NCCN and Damico’s criteria, while sPLND was performed for intermediate-risk or lower-risk patients. The surgical robots used were the da Vinci surgical system (Intuitive Surgical, Sunnyvale, CA, USA) [14] or hinotori^TM^ (Medicaroid Corporation, Kobe, Japan) [15]. The final decision to carry out ePLND depended on the surgeons or patients. Extended PLND was anatomically defined as dissection including removal of the obturator, internal iliac, and external iliac lymph nodes [4], and sPLND included the common obturator and external iliac lymph nodes. Then, patients were divided into two groups: one without pelvic lymph node metastasis (pN0) and one with pelvic lymph node metastasis (pN1). Approval of the study was obtained from the local institutional review board (Kobe University Hospital Review Board, Protocol Number: B230170, on 26 December 2023), and informed consent was obtained using an opt-out approach.

### 2.2. Choice of ML Methods

The selection of ML methods for this study was guided by their established performance and suitability for handling the characteristics of the dataset:

Logistic Regression (LR): LR, while inherently a linear model, was included in this study as a baseline for comparison. Its simplicity and interpretability make it a widely used starting point in medical research, allowing for a clear understanding of the relationship between predictors and the outcome. Additionally, the application of LASSO regularization enhances its performance by reducing overfitting and identifying the most important predictors [16]. Including LR ensures that the study provides a meaningful benchmark for evaluating the added value of non-linear ML methods, such as RF, LGBM, and SVM.

Random Forest (RF): RF is a robust ensemble learning method that builds multiple decision trees and aggregates their predictions to improve accuracy and reduce overfitting. It is particularly effective for handling structured clinical data and can model non-linear relationships and interactions between variables. RF also provides feature importance scores, which are valuable for understanding the contribution of each variable to the prediction [17].

Light Gradient-Boosting Machine (LGBM): LGBM is a gradient-boosting algorithm optimized for speed and efficiency, making it well suited for larger datasets. It sequentially builds decision trees to minimize errors, focusing on misclassified cases in each iteration. LGBM has demonstrated superior performance in various medical applications due to its ability to handle imbalanced datasets and capture complex relationships between variables [18].

Support Vector Machine (SVM): SVM is a powerful classification method that constructs a hyperplane to separate classes in a high-dimensional space. It is particularly effective for datasets where the classes are not linearly separable. Using a radial basis function kernel allows SVM to capture non-linear relationships in the data, which is essential for predicting LNI based on diverse clinical features [19].

These methods were selected not only for their popularity and demonstrated effectiveness in recent medical studies but also for their complementary strengths. For example, while LR offers interpretability, RF and LGBM excel in modeling complex interactions, and SVM is particularly robust in handling non-linear relationships. This combination ensures a comprehensive evaluation of predictive performance and allows for the identification of the most suitable approach for this clinical problem.

### 2.3. Development of ML Models

**Figure 1:**
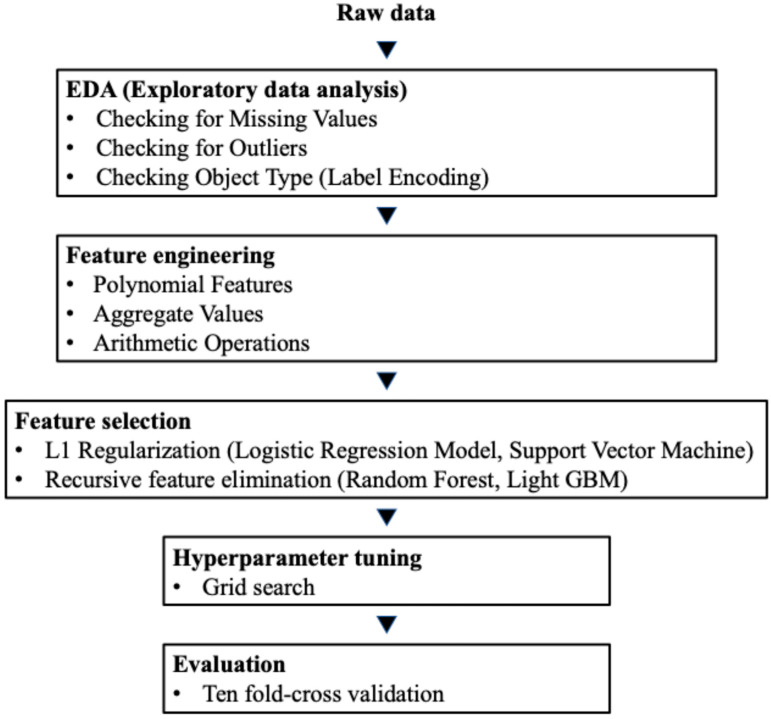
The flow diagram illustrating the overall framework of the study. AUC, area under the curve; ePLND, extended pelvic lymph node dissection; LNI, lymph node involvement; NPV, negative predictive value; PPV, positive predictive value; RARP, robot-assisted radical prostatectomy; ROC, receiver operating characteristic curve.

The procedures shown in Figure 1 were used to examine the performance of each model. In this study, nine parameters were initially prepared, including age, prostate volume, primary Gleason score (GS), secondary GS, number of positive cores, number of negative cores, PSA (ng/mL), clinical T stage, and International Society of Urological Pathology (ISUP) grading of prostate cancer. Then, feature engineering was performed, and a final set of 12 parameters was prepared. The additional features included the percentage of the positive core, product of primary and secondary GSs, and prostate-specific antigen density. These features were selected based on their clinical relevance and potential to enhance model performance. In a preliminary study, we first developed a prediction model with all 12 clinical parameters. The parameters of the LR model were reduced using LASSO regularization, whereas for the SVM, RF, and LGBM models, the best parameters were determined by the values of greedy feature selection and feature importance, which were derived using the SelectFromModel class from the feature selection module of the Python scikit-learn library [20]. The dataset was divided at a ratio of 7:3, with 70% of the data being used for machine algorithm training and hyperparameter tuning, and 30% being used for the verification set. Random splitting was performed to ensure that the distribution of key clinical features (e.g., PSA, Gleason score, and clinical stage) was similar between the training and test datasets, maintaining the representativeness of the overall cohort. In the training process for the ML algorithms, the dataset was randomly split into training and test sets at a ratio of 7:3. The training set, which included 437 patients (70% of the total), was further divided into folds for 10-fold cross-validation to tune the model and evaluate its stability. Log-loss was calculated for the validation set during each fold of the 10-fold cross-validation using the standard log-loss formula. The average log-loss across all folds was computed for each model to assess predictive performance, with lower log-loss values indicating better probability calibration and accuracy. The best hyperparameters were identified using the grid search method based on the average log-loss across the validation sets in all folds (Supplementary Table S1). All numerical variables were standardized using centering and scaling before training. The test set, which included 188 patients (30% of the total), was not used during the training or validation process. After finalizing the model with the optimal hyperparameters, its predictive performance was evaluated on the test set, which served as an independent dataset to assess the generalizability of the model.

The area under the curve (AUC), accuracy, F1 score, specificity, positive predictive value (PPV), and negative predictive value (NPV) of each model were comprehensively evaluated to compare performance between the models. To evaluate the performance of the developed model, we used bootstrapping with 1000 iterations. The AUC scores were calculated for each bootstrap sample. The resulting 95% confidence interval (CI) for the AUC was calculated. This ensured that our model’s performance was robustly estimated and provided a reliable measure of its discriminative ability. In addition, we used a decision curve analysis (DCA) to evaluate the clinical net benefit of the models [17]. DCA evaluates the clinical utility of prediction models by considering the net benefit across a range of risk thresholds. The threshold in DCA is a probability value, which determines the point at which the expected benefit of a treatment is equal to the expected harm of avoiding the treatment. Models with higher AUC and net clinical benefit were considered superior models.

### 2.4. Feature Importance Measurement

For the RF and LGBM algorithms, the total gain was used to measure the relative importance of the clinical features. The gain is the relative contribution of a feature to the model, calculated by taking each feature’s contribution to each tree in the gradient-boosting decision tree model. In both models, the features with higher gain are more important for generating the prediction. The prediction accuracy of each model was further verified by the decision curve analysis.

### 2.5. Statistical Analysis

The Wilcoxon rank-sum test was used for comparing continuous variables between the pN0 and pN1 groups, as these variables did not meet the assumption of normality required for the *t*-test, and the Wilcoxon test is robust to outliers. For categorical variables, Fisher’s exact test was applied in all cases, as some categories had fewer than 10 observations. Fisher’s exact test ensures statistical validity when sample sizes in specific categories are small. *p*-Values lower than 0.05 were considered statistically significant. Corresponding 95% confidence intervals (95% CIs) were calculated for variables. The modeling process was implemented using scikit-learn version 1.2.2 in Python (version 3.10.12, Python Software Foundation, Beaverton, OR, USA).

## 3. Results

### 3.1. Study Participants

**Figure 2:**
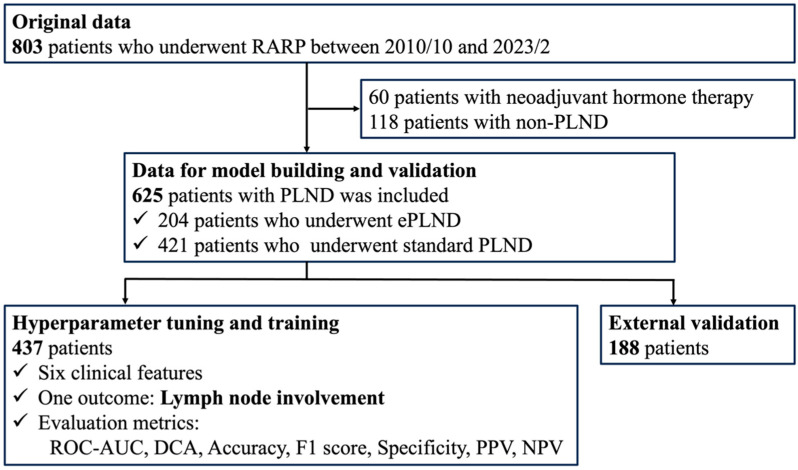
The development process of the machine learning model. The process from feature engineering to evaluation was repeated many times to improve the model’s accuracy.

The model development and validation flow diagram is presented in Figure 2. A total of 625 patients were included in the current study, including those who underwent ePLND and sPLND. The baseline characteristics of the included patients are shown in Table 1. Overall, 34 patients (5.4%) had LNI. The PSA, clinical stage, number of positive cores, percentage of positive cores, overall ISUP grade, pathologic stage, and resection margin also differed significantly between patients with pN0 and pN1 disease (Table 1).

### 3.2. Final Features of Each Model

From the initial set of six features, the features of the RF and LGBM model were reduced to the four features of the “clinical T stage”, “PSA”, “percentage of positive cores”, and “age” using the RFE. This reduction was the result of tuning to achieve higher accuracy on the training set. Therefore, hyperparameter tuning was conducted using models with four features for RF and LGBM, and six features for LR and SVM (Table 2).

### 3.3. Comparison of ML Models with Conventional Nomograms

**Figure 3:**
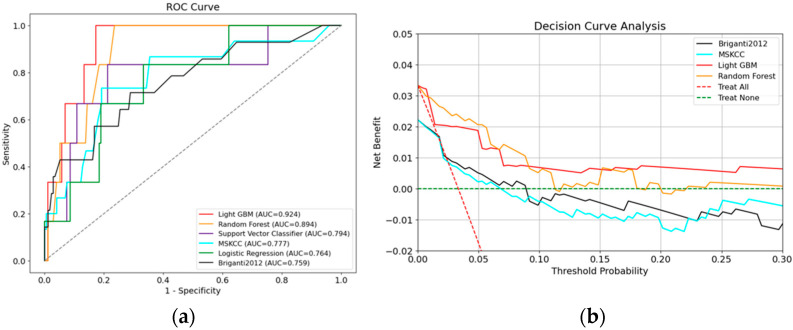
(**a**) The AUCs of all ML models. LGBM and RF showed particularly high AUCs compared to conventional nomograms. AUC, area under the curve; ML, machine learning; LGBM, Light GBM; RF, Random Forest. (**b**) DCA of LGBM, RF, and a conventional nomogram for predicting LNI. The y-axis measures the net benefits, and the x-axis is the risk threshold. LGBM and RF, which showed high AUCs, had net benefits significantly higher than conventional nomograms at all thresholds. LGBM demonstrated high and stable net benefit at intermediate threshold probabilities (0.10 to 0.30), while the RF model exhibited a high net benefit at low threshold probabilities, particularly within the range of 0.02 to 0.08. DCA, decision curve analysis; LGBM, Light GBM; LNI, lymph node involvement; RF, Random Forest.

The AUC for each model is shown in Table 2 and Figure 3a. The results demonstrating the model’s performance were all evaluated using the validation dataset. The prediction results show that the LGBM model had the highest AUC of 0.924, followed by the RF (0.894) model. The DCA revealed that the LGBM and RF offer substantial net benefits, with certain nuances in their performance across different threshold ranges. LGBM demonstrated the highest net benefit at low threshold probabilities (≤0.05). The model maintained a high and stable net benefit at intermediate threshold probabilities (0.05 to 0.15) and did not show a significant decline in net benefit at higher threshold probabilities (0.15 to 0.30). Similarly, the RF exhibited a high net benefit at low threshold probabilities, particularly within the range of 0.02 to 0.04. At intermediate threshold probabilities, RF maintained a comparable net benefit to LGBM. At higher threshold probabilities, the net benefit of RF slightly decreased but remained positive in most cases. Feature importance was assessed using a function of the LGBM and RF algorithms, which achieved high predictive performance on the test data. 

**Figure 4:**
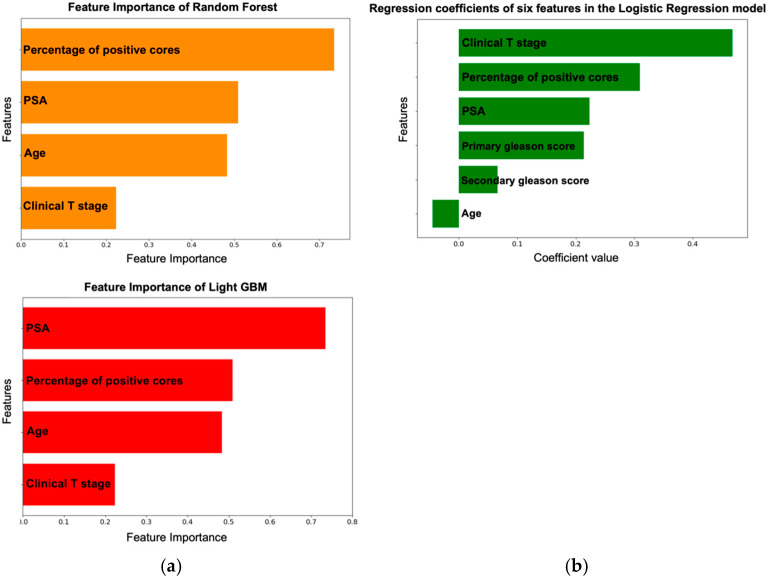
(**a**) Importance values of the clinical factors measured using the total gain of the RF and LGBM algorithms. LGBM, Light GBM; RF, Random Forest. (**b**) Regression coefficients of features in the LG model. LG, Logistic Regression.

The findings of this assessment indicated that the percentage of positive cores and PSA were important features (Figure 4a)**.** Conversely, clinical T stage showed the highest regression coefficients of the seven features in the LR model (Figure 4b).

The 0.05, 0.10, and 0.20 thresholds of the LR, RF, and LGBM models, and Briganti 2012 and MSKCC nomograms, were used to test their clinical utility for guiding treatment options (Table 3). The LGBM model demonstrated higher accuracy than the other models, with a PPV of 25.0% (F1 score: 36.4, specificity: 93.1%) at a 0.05 threshold, and a PPV of 22.2% (F1 score: 26.7, specificity: 96.0%) at a 0.10 threshold. At a 0.15 threshold, the LGBM and LR showed high accuracy with a PPV of 28.6% (F1 score: 30.8, specificity: 97.1%), and 20.0% (F1 score: 17.4, specificity: 97.7%).

## 4. Discussion

In this study, we developed ML models for predicting LNI in Japanese patients with prostate cancer, using clinically relevant factors in a well-structured approach. 

Research on the accurate prediction of LNI in patients with prostate cancer has been actively conducted for some time. Among the developments, the 2012 Briganti nomogram is the most renowned, and its applicability to Japanese patients has also been validated [21,22,23,24]. This model was updated in 2019 to include findings from multiparametric magnetic resonance imaging (mpMRI). Preoperative mpMRI can predict nodal metastases in patients with prostate cancer, potentially allowing a better selection of those candidates requiring ePLND [25,26,27]. However, studies indicate that this latest 2019 version does not outperform previous nomograms [28,29], and may lack flexibility. Additionally, there are reports of improved accuracy by incorporating Prostate-specific Membrane Antigen (PSMA) Positron Emission Tomography into the nomogram [30,31,32,33]; however, PSMA’s limited availability raises concerns about widespread clinical implementation.

Recently, there has been an increase in studies utilizing ML models. One of the superior aspects of ML models is their higher flexibility compared to traditional models, along with their ability to achieve high accuracy when trained with sufficient data. Unlike traditional models, ML algorithms can effectively handle complex, non-linear relationships between variables and are less reliant on assumptions such as linearity or independence. This capability makes them particularly advantageous for capturing intricate interactions in clinical datasets. Moreover, while the current study focuses on structured clinical data, ML methods have the potential to incorporate unstructured data types, such as imaging or text, in future iterations. This adaptability underscores the scalability and long-term potential of ML models in clinical applications [34]. Additionally, the developed models can be output as applications allowing for immediate use in actual clinical settings. As more data accumulate, it becomes easy to update the model regularly. If the database used as training data is large and diverse, the utility of the model is likely to be further enhanced. Recent studies have further highlighted the advantages of ML models in clinical applications. For instance, Kraujalis et al. demonstrated the utility of ML models in predicting mortality risks in prostate cancer, emphasizing their ability to improve prognostic accuracy by integrating diverse clinical parameters [35]. Similarly, Subrahmanya et al. discussed the broader role of ML models in healthcare, including their potential to transform patient care through personalized medicine and data-driven decision making [36]. These findings align with our results, which illustrate the adaptability and clinical relevance of ML models. While prior studies have highlighted the benefits of ML models over conventional nomograms for LNI prediction in prostate cancer, our approach further maximizes these models’ potential through systematic feature engineering and optimization. As illustrated in Figure 1, our study introduced key methodological enhancements, which distinguished it from previous work. Our methodology involved several key steps, beginning with an Exploratory Data Analysis (EDA), in which we rigorously examined the dataset to identify missing values, and outliers, and ensure data integrity. Following this, we conducted feature engineering by creating polynomial and aggregate features alongside conventional clinical variables, capturing complex data relationships to potentially boost model performance. Next, feature selection was applied using techniques such as L1 regularization in Logistic Regression and recursive feature elimination in Random Forest and Light GBM, allowing us to isolate relevant predictors and enhance model interpretability [37,38]. Finally, we optimized model parameters through grid searching, ensuring that each algorithm performed at its best. This structured approach offers insights into ML’s capabilities for LNI prediction in prostate cancer, potentially supporting more accurate risk stratification and treatment planning. In the RARP procedures included in this study, both hinotor and da Vinci surgical systems were used. The hinotor and da Vinci surgical systems share similar operational features and surgical techniques, ensuring consistency in procedures. Previous studies have shown that perioperative outcomes of RARP using hinotor were comparable to those using the da Vinci system, with no significant differences in key outcomes such as operative time and positive surgical margins [15]. Therefore, the influence of the surgical system on the results of this study is considered minimal. Sabbagh et al. [39] developed an ML model for predicting LNI in German and American cohorts, and it outperformed the Roach formula, the MSKCC calculator, and the Briganti 2012 nomogram. Wei et al. [40] developed an ML model based on big data that could reduce the number of ePLND procedures by approximately 50%. However, their studies used the same features for all ML models or omitted feature engineering, thus underutilizing model-specific strengths. Furthermore, none of these studies utilized datasets specific to Japanese patients, limiting their applicability to populations with potentially different clinical, biological, or demographic characteristics. Hou et al. [41] demonstrated that adding mpMRI features to an ML model enhanced performance, with results aligned with our study’s patient cohort. While standard PLND can miss some LNI cases, the enhanced flexibility of ML models supports practical applications. Although Goto et al. [23] developed a nomogram to identify the candidates for ePLND for Japanese patients and evaluated its usefulness, our study is the first to attempt to use ML to identify candidates for ePLND.

In this study, we examined the utility of the ML model from various aspects, including AUC and DCA. Evaluating both AUC and DCA is crucial as they provide complementary insights into model performance. While AUC measures the overall ability of the model to distinguish between positive and negative cases, offering a general sense of discrimination power, DCA assesses the clinical utility by quantifying the net benefit across different threshold probabilities. This dual evaluation allows for a more comprehensive understanding of how the model performs not only in theoretical terms but also in practical, real-world scenarios. The AUCs around 0.9 indicate high accuracy for RF and LGBM. Although F1 scores were modest, likely due to outcome imbalance (only 5.4% of patients had LNI), both RF and LGBM surpassed traditional models in F1 scores, suggesting improved prediction specificity. Traditional nomograms, such as Briganti and MSKCC, often overestimate LNI risk, which could lead to unnecessary ePLND. DCA showed that both LGBM and RF are robust models that provide significant clinical benefits across various threshold probabilities. LGBM shows a slight edge, particularly at lower threshold probabilities, making it an excellent choice for early decision-making scenarios (Figure 3b). Overall, both models demonstrate strong potential for clinical application, with LGBM having a slight advantage in terms of net benefit across most threshold probabilities. These results indicate that the ML model tends to diagnose patients with LNI, and this is consistent with the results of previous studies [40]. 

In the feature importance analysis, RF and LGBM highlighted positive core percentages and PSA levels as top predictors, while T stage emerged as significant in the Logistic Regression model. Despite initially exploring new features, we achieved high AUC scores with a minimal set of features similar to those used in the Briganti 2012 nomogram, even improving accuracy by reducing features from six to four. This simplification suggests that some omitted variables, such as GS, may not be critical in predicting metastasis. Our results emphasize the value of non-linear models like RF and LGBM for improving LNI prediction accuracy in prostate cancer. 

Several limitations of our study should be noted. First, because of the small number of cases and the study being conducted at a single institution, it is not possible to ascertain the models’ utility on data from other facilities. The lack of diversity in patient populations limits the generalizability of our findings, particularly for populations with different clinical practices or demographics. Large-sized domestic datasets are necessary to develop and validate a practical predictive model for Japanese patients. Second, our study was conducted using retrospective data, and is therefore susceptible to biases inherent to such studies. These include missing data, inconsistent reporting, and incorrect data entry. Additionally, the study period spans over a decade (2010–2023), during which changes in clinical practices or surgical techniques may have introduced variability into the dataset. Time-stratified analyses could be considered in future studies to address such temporal effects. Finally, the preoperative decision-making criteria for performing ePLND depended on the NCCN risk classification. This guideline for ePLND may miss some metastatic lymph nodes. Including cases with sPLND could also result in selection bias, as it may miss some metastatic lymph nodes, as positive lymph nodes can be located outside the routine surgical template. Future studies should adopt standardized lymph node dissection protocols across institutions to minimize these potential biases.

## 5. Conclusions

Applying ML-based models offers a promising strategy for enhancing surgical decision making in prostate cancer and could improve long-term patient outcomes. Our findings suggest that ML models provide a more precise approach to identifying candidates for ePLND, potentially advancing prostate cancer management.

## Figures and Tables

**Table 1:** Patient characteristics.

	pN0 *n* = 591 (94.6%)	pN1 *n* = 34 (5.4%)	*p*-Value
Median age, yr (IQR)	68.3 (64.0–72.4)	70.5 (65.1–73.1)	0.35
Median BMI (IQR)	24.0 (22.0–25.0)	24.0 (22.0–26.0)	0.528
PSA, ng/ml (IQR)	8.0 (5.6–11.7)	12.0 (7.0–19.0)	0.002
cT stage, *n* (%)			<0.001
cT2a	294 (49.8%)	11 (32.4%)	
cT2b	20 (3.4%)	0	
cT2c	126 (21.3%)	5 (14.7%)	
cT3a	83 (14.0%)	14 (41.2%)	
cT3b	10 (1.7%)	3 (8.82%)	
cT4	0	1 (2.94%)	
Median P-volume, ml (IQR)	27.0 (20.0–37.0)	30.0 (21.5–43.0)	0.223
Median cores, *n* (IQR)	12 (10–12)	12 (11–12)	
Median positive cores overall, *n* (IQR)	3.0 (2.0–5.0)	5.0 (3.3–7.8)	<0.001
Median percentage of positive cores overall, % (IQR)	30.0 (17.0–50.0)	48.0 (33.0–67.0)	<0.001
ISUP overall, *n* (%)			0.016
1	62 (10.9%)	3 (8.82%)	
2	178 (30.1%)	3 (8.82%)	
3	125 (21.2%)	6 (17.7%)	
4	182 (30.8%)	16 (47.1%)	
5	44 (7.45%)	6 (17.7%)	
pT, *n* (%)			<0.001
pT0	1 (0.2%)	0	
pT2a	192 (32.5%)	2 (5.9%)	
pT2b	23 (3.9%)	2 (5.9%)	
pT2c	197 (33.3%)	4 (11.8%)	
pT3a	131 (22.2%)	13 (38.2%)	
pT3b	36 (6.1%)	13 (38.2%)	
pT4	2 (0.3%)	0	
N/A	9 (1.5%)	0	
Resection margin, *n* (%)	47 (8.0%)	11 (32.4%)	<0.001

PSA, prostate-specific antigen.

**Table 2:** Prediction results on the validation dataset evaluated by repeated ten-fold cross-validation and sorted by ROC-AUC.

Model	Features	AUC	95%CI
Light GBM	cT stage, PSA, Positive cores %, Age	0.924	[0.850, 0.982]
Random Forest	cT stage, PSA, Positive cores %, Age	0.894	[0.815, 0.974]
Support Vector Machine	cT stage, PSA, Positive cores %, Age, Primary GS, Secondary GS	0.794	[0.513, 0.957]
MSKCC	cT stage, PSA, Positive cores %, Age, Primary GS, Secondary GS	0.771	[0.615, 0.895]
Logistic Regression	cT stage, PSA, Positive cores %, Age, Primary GS, Secondary GS	0.764	[0.561, 0.930]
Briganti, 2012	cT stage, PSA, Positive cores %, Primary GS, Secondary GS	0.759	[0.597, 0.897]

ROC-AUC, Area under the receiver operating characteristic curve; GS, Gleason score; CI, Confidence interval.

**Table 3:** Diagnosis and clinical implications according to treatment option.

Treatment Option	Accuracy (%)	F1 Score	Specificity (%)	PPV (%)	NPV (%)
5% Cutoff					
MSKCC	21.9	16.6	18.6	9.1	97.0
Briganti 2012	35.3	17.7	30.6	9.8	98.2
Random Forest	75.6	21.4	74.7	12.0	100.0
Light GBM	92.2	36.4	93.1	25.0	98.8
Logistic Regression	68.9	30.8	69.0	6.9	98.4
Support Vector Classifier	3.3	6.5	0.0	3.3	N/A
10% Cutoff					
MSKCC	34.8	20.1	36.0	11.3	98.4
Briganti 2012	55.1	20.8	53.2	12.0	96.8
Random Forest	83.3	21.1	83.9	12.5	98.7
Light GBM	93.9	26.7	96.0	22.2	97.7
Logistic Regression	89.4	12.5	91.4	11.8	97.6
Support Vector Classifier	12.8	7.1	9.8	3.7	100.0
15% Cutoff					
MSKCC	45.5	23.0	50.6	13.3	97.8
Briganti 2012	70.1	26.3	69.9	16.1	96.8
Random Forest	89.4	24.0	90.8	15.8	98.1
Light GBM	95.0	30.8	97.1	28.6	97.7
Logistic Regression	95.0	17.4	97.7	20.0	97.1
Support Vector Classifier	59.4	12.1	58.6	6.5	99.0

PPV, Positive predictive value; NPV, Negative predictive value.

## Data Availability

The datasets generated and analyzed during the current study are available from the corresponding author upon reasonable request. Access to the data may be subject to ethical and confidentiality considerations.

## Supplementary Materials

The following supporting information can be downloaded at: https://www.mdpi.com/article/10.3390/cancers16234073/s1. Supplemental Table S1. Optimized Hyperparameters and Performance Scores for Machine Learning Models Used in Lymph Node Metastasis Prediction.
