# Supplementary material for: Utility of Machine Learning Models to Predict Lymph Node Metastasis of Japanese Localized Prostate Cancer
Source: Cancers (Basel). 2024 Dec 5;16(23):4073. doi: 10.3390/cancers16234073 (PMC11640458; doi:10.3390/cancers16234073)
Supplement: Supplementary file 1 [file cancers-16-04073-s001.zip › cancers-3329512-supplementary.pdf]

Table S1. Optimized Hyperparameters and Performance Scores for Machine Learning Models Used in Lymph Node Metastasis Prediction".

Model	Hyperparameter	Value
**LightGBM**	max_depth	3
	num_leaves	2
	reg_alpha	0.03
**Random Forest**	max_depth	2
	min_samples_leaf	4
	min_samples_split	10
	n_estimators	500
**Logistic Regression**	model__C	0.1
	model__class_weight	None
	model__penalty	l2
**SVM**	model__C	0.001
	model__gamma	0.001
	preprocessing	StandardScaler()
